# Development of Neurological Mouse Model for Toxoplasmosis Using* Toxoplasma gondii* Isolated from Chicken in Kenya

**DOI:** 10.1155/2017/4302459

**Published:** 2017-05-24

**Authors:** John Mokua Mose, David Muchina Kamau, John Maina Kagira, Naomi Maina, Maina Ngotho, Adele Njuguna, Simon Muturi Karanja

**Affiliations:** ^1^Department of Medical Laboratory Science, School of Medicine and Health Sciences, Kenya Methodist University, P.O. Box 45240-00100, Nairobi, Kenya; ^2^Department of Public Health, Jomo Kenyatta University of Agriculture and Technology (JKUAT), P.O. Box 62000-00200, Nairobi, Kenya; ^3^Department of Animal Sciences, Jomo Kenyatta University of Agriculture and Technology (JKUAT), P.O. Box 62000-00200, Nairobi, Kenya; ^4^Department of Biochemistry, Jomo Kenyatta University of Agriculture and Technology (JKUAT), P.O. Box 62000-00200, Nairobi, Kenya; ^5^Department of Animal Health & Production, Mount Kenya University, P.O. Box 342-01000, Thika 342-01000, Kenya

## Abstract

Animal models for the toxoplasmosis are scarce and have limitations. In this study, a neurological mouse model was developed in BALB/c mice infected intraperitoneally with 15 cysts of a* Toxoplasma gondii* isolate. The mice were monitored for 42 days and euthanized at different time points. Another group of mice were orally treated with dexamethasone (DXM: 2.66 mg/kg daily, 5.32 mg/kg daily) at 42 days after infection and monitored for a further 42 days. A mortality rate of 15% and 28.6% was observed in mice given 2.66 mg/kg/day and 5.32 mg/kg/day of DXM, respectively. The mean cyst numbers in the brain of DXM treated mice increased up to twofold compared with chronically infected untreated mice. Infections up to 42 days were associated with an increase in both IgM and IgG levels but following dexamethasone treatment, IgM levels declined but IgG levels continued on rising. The brain of toxoplasmosis infected mice showed mononuclear cellular infiltrations, neuronal necrosis, and cuffing. The severity of pathology was higher in mice treated with dexamethasone compared to the positive control groups. The findings of this study demonstrate that DXM-induced reactivation of chronic toxoplasmosis may be a useful development of laboratory animal model in outbred mice used for* in vivo* studies.

## 1. Introduction

Toxoplasmosis, caused by* Toxoplasma gondii, *is one of the most common parasitic infections of man and other warm-blooded animals [1]. It is estimated that between 500 million to 2 billion people worldwide are chronically infected with this parasite [2]. In Kenya, a prevalence between 23% and 60% has been described in the local human population [3]. Toxoplasmosis in immunocompetent individuals are asymptomatic with a few cases being clinical. However, the parasite can cause severe disease in people with immunodeficiencies and in fetuses infected in utero [2]. These two classes of patients present different challenges to health professionals in terms of diagnosis, case management, and drug treatment. Following infection, there is brief acute stage characterized by the proliferative tachyzoite stage of the parasite, but thereafter the parasite undergoes latency, characterized by slowly growing bradyzoites within tissue cysts. The tissue cysts remain viable presumably during the life of the host. During infections, the parasite invades a variety of immune cells and is subsequently disseminated throughout the body, traversing biological barriers to reach immunologically privileged sites such as the brain where it can cause severe pathologies [4, 5].

Both humoral and cell mediated immunity perform a role in resistance against* T. gondii *[6]. In human, immunosuppression may result in reactivation of a latent infection following cyst rupture and renewed parasite proliferation and dissemination throughout the body. Such* T. gondii *reactivation in HIV/AIDS patients results in life-threatening toxoplasmic encephalitis and associated neuropsychiatric manifestations [7]. Changes in the levels of antibodies have been documented following the reactivation of* T. gondii *infection. In one study [8], the specific IgG and IgM titers of the toxoplasmosis/dexamethasone-treated mice were depressed significantly after dexamethasone treatment, but whether these changes are diagnostic has not been well ascertained.

Disease development is influenced by other factors, the route of infection, host, and strain of parasite [9]. The existing mice murine models have used* T. gondii* strains from other continents [10, 11] with none describing the pathogenesis of strains isolated from Africa. In a commonly used SCID mouse model of* T. gondii*, sulfadiazine treatment has been shown to suppress infection thus allowing chronicity to develop, and its withdrawal leads to disease relapse [10]. SCID mice have some limitations such as nonavailability in most laboratories and cumbersome to work with because of their severely impaired immune systems and the requirement for them to be kept in sterile conditions to prevent opportunistic infections [10]. On the other hand, mice such as BALB/c are widely available and used as models to study different diseases [11]. The aim of the current study was to investigate the changes in immunoglobulin (IgG and IgM) levels and brain histopathology in an immunosuppressed BALB/c rodent model for toxoplasmosis.

## 2. Materials and Methods

### 2.1. Ethical Clearance

Prior to commencement of the study, all protocols and procedures used were reviewed and approved by the Institutional Animal Care and Use committee of the Institute of Primate Research in Kenya (approval number: IRC/21/11).

### 2.2. Laboratory Animals

Female BALB/c white mice were obtained from the rodent breeding facility at the Institute of Primate Research, Nairobi, Kenya. They were 6–8 weeks old and weighed 20–30 g. They were housed under standard laboratory conditions, in plastic cages (medium size cages; length 16.9 inches, width 10.5 inches, and height 5 inches) and were provided with wood shaving bedding and nesting material. Food (Mice Pellets®, Unga Feeds Ltd., Kenya), and drinking water was given ad libitum.

### 2.3. *Toxoplasma gondii* Isolate and Expansion

The* T. gondii* isolate used in this study was obtained from the brain of free range chicken from Thika region, Kenya. Briefly, the chicken was sacrificed by cervical dislocation and the brain tissue collected under sterile conditions. The brains were ground in a mortar using pestle; 1 mL of phosphate buffered saline (PBS, pH 7.2) was added and homogenized using tissue homogenizer [12]. Enumeration of cysts was done by transferring three aliquots of 20 *μ*L of the brain suspensions onto microscopic slides; a coverslip was placed over each sample and the number of cysts counted in the entire sample under 20x magnifications directly without staining. The brain suspension was serially diluted with PBS to adjust to a desired final concentration of 15 tissue cysts/200 *µ*l [13]. To obtain cysts for experimental infections, three BALB/c mice were intraperitoneally injected each with 15 tissue cysts to allow for expansion of* T. gondii* cysts for use in experimental infection described below. The mice were monitored for six weeks after infection and anesthetized using concentrated CO_2_ gas before sacrifice and parasites were isolated as stated above.

Presence of* Toxoplasma gondii *in the chicken and mice samples was confirmed by extracting DNA from brain samples using a Quick-gDNATM MiniPrep kit (Zymo research, USA; Catalogue No. D3025) and amplification of the 529 bp repeat units was carried out by nested PCR as previously described [14, 15]. Secondary amplification products were electrophoresed on 1.5% agarose gel stained with ethidium bromide and visualized under ultraviolet (UV) light.

### 2.4. Drug

The immunosuppressive drug used in this study was dexamethasone (DXM) (DecadronDexPak, PHARMA Links, India). The drug was administered orally in drinking water at concentrations of 4 mg/L and 8 mg/L. At these concentrations, the dosing is equivalent to 2.66 and 5.32 mg/kg daily, respectively, assuming that the average weight of a mouse is 30 g and that the water consumption rate is 10 mL daily [16].

### 2.5. Experimental Design

#### 2.5.1. Sampling and Sample Size Determination

The experimental mice were assigned to the experimental groups through simple random sampling technique. The sample size of the study subjects was estimated based on a previous study that yielded statistically analyzable (conclusive) results [17]. A total of 80 BALB/c mice were intraperitoneally administered with 15* T. gondii* cysts in a 200 *µ*l inoculum [18]. Another eight mice were used as uninfected controls.

#### 2.5.2. Acute and Chronic Infection Study

In the first part of the experiment, 32 mice in groups of four were randomly chosen and euthanized in batches of four by concentrated CO_2_ inhalation on 3, 5, and 7 dpi for acute infection and 14, 21, 28, 35, and 42 dpi for chronic infection. Four BALB/C mice were controls and not infected with* T. gondii.*

After euthanasia, sampling for blood from the heart was done as previously described [19]. The blood was kept in separate labelled microfuge tubes for each mouse, on the bench till a blood clot was formed, and then kept at 4°C overnight. The clots were then disturbed using wooden splints and centrifuged in a microfuge (Eppendorf Centrifuge Model 5415) at room temperature for 3 minutes at 200*g*. The serum was transferred into other respective labelled tubes and stored at −20°C to be used for detecting antibodies (IgG and IgM) by ELISA (Mabtech AB, Sweden), according to the manufacturer's instructions. The brain tissue was also collected and divided in halves; one half was processed to prepare tissue homogenates for cyst enumeration, while the other half was preserved in 10% formalin and used for histology as described below.

#### 2.5.3. Determination of Effect of Dexamethasone

In the second part of the experiment, 48 previously infected BALB/c mice were obtained at 42 dpi and assigned into three groups. Sixteen mice per group were each administered with dosages of 2.66 mg/kg/day (Group 1) and 5.32 mg/kg (Group 2) daily of dexamethasone (DXM) in drinking water over a period of 6 weeks [17]. Another sixteen infected nontreated mice were used as controls (Group 3). Four uninfected control mice were given untreated water (Group 4). The mice were monitored daily over six weeks for survival analysis and any clinical signs and mortalities were recorded. After every two weeks for six weeks after treatment, four mice from each group were serially euthanized using concentrated carbon dioxide and sampling done as previously described in Section 2.5.2. Mice that showed any severe clinical signs of toxoplasmosis were anesthetized immediately using concentrated carbon dioxide and sampling of blood and brain was also done.

### 2.6. Quantification of Brain Cysts

As described earlier, one half of the brain from each mouse in the experimental infections was used for evaluation of cyst burden. The number of cysts was determined by placing three aliquots of 20 *µ*l of the brain suspensions onto microscopic slides and counting was done as previously described [13]. Using the average of the counts, the concentration of cysts per ml of brain suspension was calculated.

### 2.7. Quantification of Parasite-Specific IgG and IgM Antibodies

Serum was prepared from the blood collected from the heart. Antibody titers for IgG and IgM were estimated using commercial ELISA kits (Mabtech AB, Augustendalsvagen 19, Sweden). Each well of a high protein binding 96-well plate (Maxisorp) was coated with 100 *µ*l/well of anti-IgG/IgM antibody diluted to 1 *μ*g/ml in PBS and incubated overnight at 4°C. The plates were washed twice with PBS (200 *µ*l/well) and blocked by adding 200 *µ*l/well of PBS with 0.05% Tween 20 containing 0.1% BSA (incubation buffer) and incubated for 1 hour at room temperature. After washing five times with PBS-Tween, 100 *μ*l of serum samples or recombinant mouse IgG and IgM standards was then added to the plates and incubated for 2 hrs at 37°C. After washing, 100 *μ*l per well of anti-IgG-ALP diluted 1 : 1000 and anti-IgM-ALP diluted 1 : 500 in incubation buffer was added, respectively, and incubated for 1 hour at room temperature. After washing, 100 *µ*l of p-nitrophenyl phosphate substrate was added to each and after a suitable developing time, the optical density (OD) of duplicated samples was measured at 405 nm for pNPP using a spectrophotometer (Titertek Multiscan ELISA reader, Helsinki, Finland) and compensated by comparing the OD of a standard positive serum in each plate. The ELISA indexes of 0.2 were determined as positive cut-off values.

### 2.8. Histological Analysis

Brain tissues were fixed in 10% buffered formalin and processed by paraffin embedding and sectioning. To verify the histological changes, tissue sections were stained with haematoxylin and eosin and observed under light microscope. The severity of the histopathological lesions was evaluated by grading the lesions using a random scale as described by Tanaka et al. [20]. All of the histological analysis was done using a 40x objective. The scores for all lesions were added for each mouse, and the total pathological score for each mouse was used for data analysis.

### 2.9. Statistical Analysis

The results were entered into MS Excel program (Microsoft, USA) before being exported to GraphPad prism version 5.0 (GraphPad Software, USA) for statistical analysis. Survival of mice on different drug regimens was evaluated by the Kaplan-Meier product limit method. The difference between the curves obtained was analysed by the Wilcoxon rank sum test. Differences in the cyst numbers, relative IgG, and IgM titers between groups were examined by Student's* t*-test. The level of statistical significance was set at 0.05.

## 3. Results

### 3.1. Clinical Signs and Cumulative Survival

The infected mice treated with dexamethasone [2.66 mg/kg/day and 5.32 mg/kg/day] showed various clinical signs which increased with increase in dosage level. They included tottering gait with decreased activity coupled with piloerection, tachypnea, hunched appearance, and evidence of emaciation. Mice infected and not treated did not show any clinical signs. The uninfected control mice did not manifest any clinical signs.

**Figure 1:**
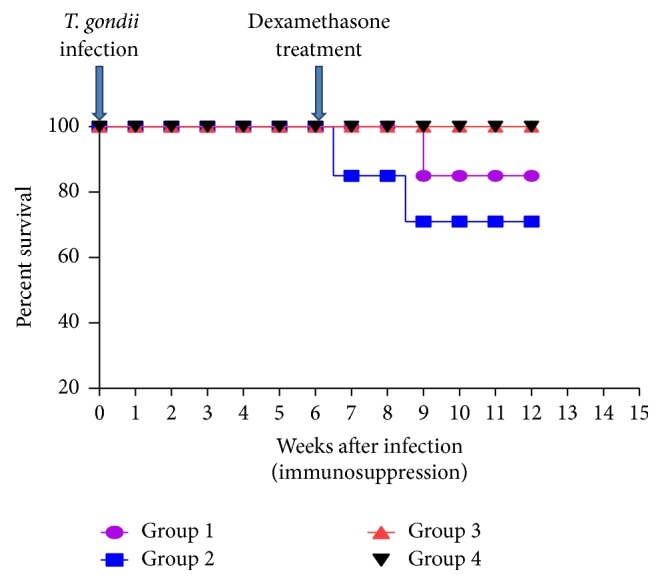
Effect of dexamethasone on survival (Kaplan-Meier estimates) of mice infected with* T. gondii* infection and treated with dexamethasone: Group 1 = mice infected with* T. gondii* and treated with dexamethasone at 2.66 mg/kg/day; Group 2 = mice infected with* T. gondii* and treated with dexamethasone at 5.32 mg/kg/day; Group 3 = mice infected with* T. gondii* and not treated with dexamethasone; Group 4 = noninfected control.

Results of the cumulative survival of mice evaluated using the Kaplan-Meier method showed that (100%) both those noninfected and those infected and not treated survived during the whole experimental period (Figure 1). However, a mortality rate of 15% and 28.6% was recorded in mice infected and treated with 2.66 mg/kg/day and 5.32 mg/kg/day, respectively. Treatment with 5.32 mg/kg daily of DXM significantly (*P* < 0.05) increased the mortality rate as compared to infected untreated mice. In this study, 85% of mice treated with 2.66 mg/kg/day of dexamethasone survived after the fourth week after treatment, while the mice treated with 5.32 mg/kg/day of dexamethasone showed 85% survival at third week after treatment, decreasing to 71.4% survival at week 9 after treatment. The percentage survival time in days of the* Toxoplasma* infected treated groups was significantly (*P* < 0.05) lower with increase in dosage level.

### 3.2. Brain Tissue Cysts Burden

**Figure 2:**
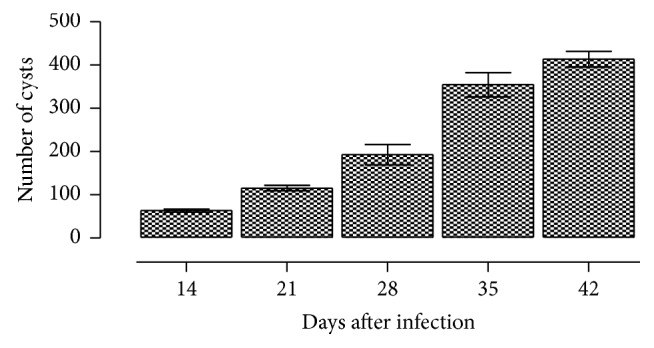
Number of cysts per ml of brain tissue (mean ± SD) of mice with established chronic toxoplasmosis.

Presence of* Toxoplasma* tissue cysts in the brain of infected nontreated mice was observed from 14 dpi and was more evident as the disease progressed. The mean cyst numbers ranged from 63.5 ± 6.758 (mean ± SD) at 14 dpi and increased to 115.8 ± 12.53; 192.8 ± 47.23; 345 ± 56.42; and 416.3 ± 32.2 [*P* < 0.05] at 21, 28, 35, and 42 dpi, respectively (Figure 2).

**Figure 3:**
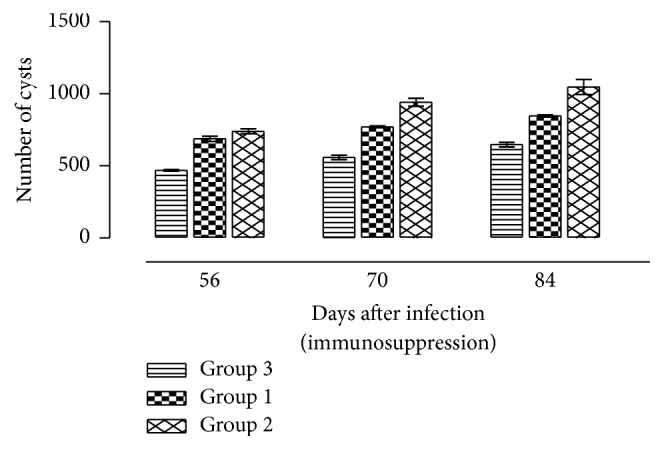
Number of cysts per ml of brain tissue (mean ± SD) of mice with established chronic toxoplasmosis after treatment with 2.66 mg/kg/day and 5.32 mg/kg/day of dexamethasone: Group 1 = mice infected with* T. gondii* and treated with dexamethasone at 2.66 mg/kg/day; Group 2 = mice infected with* T. gondii* and treated with dexamethasone at 5.32 mg/kg/day; Group 3 = mice infected with* T. gondii* and not treated with dexamethasone.

After treatment with DXM, a progressively increasing number of tissue cysts were observed in the brains of mice in various treatment groups (Figure 3). In the mice treated with 2.66 mg/kg/day, the mean number of tissue cysts per ml of brain sample at 14, 28, and 42 days post treatment (dpt) was 687.99 ± 35.43, 770.495 ± 12.722, and 845.88 ± 15.43, respectively. For the mice treated with 5.32 mg/kg/day, there was significant increase in cyst numbers, with the mean numbers of cysts per ml of brain sample at 14, 28, and 42 dpt being 740.17 ± 34.6, 940.78 ± 54.7, and 1047.14 ± 103.76, respectively The mean number of tissue cysts per ml of brain sample in the infected nontreated mice at 14, 28, and 42 dpt was 468.91 ± 11.35, 559.42 ± 30.41, and 647.39 ± 32.12, respectively. The tissue cyst numbers per ml of brain sample in the mice treated with 2.66 mg/kg/day and 5.32 mg/kg/day were significantly (*P* = 0.001) higher than those from the infected nontreated mice. However, the differences in numbers of cysts in the group treated with 2.66 mg/kg/day and 5.32 mg/kg/day were not significant (*P* > 0.05).

**Figure 4:**
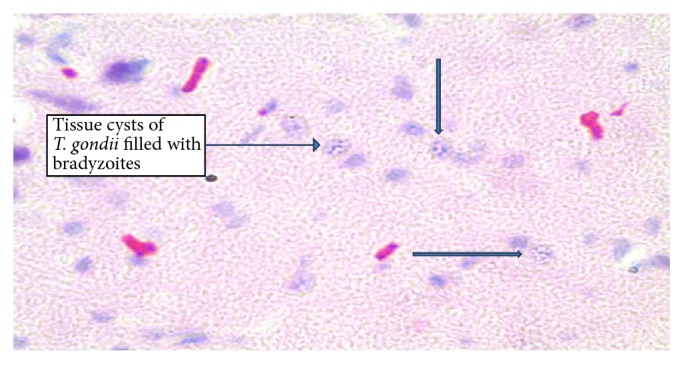
*Toxoplasma gondii* cysts in the brains of chronically infected BALB/c mice.

*T. gondii* cysts were observed in the H&E stained histological sections. These cysts were round in shape, were variable in size, and were widely dispersed throughout the tissue (Figure 4). The proportion of mice having* T. gondii* tissue cysts in the mice treated with 2.66 mg/kg/day and 5.32 mg/kg/day of DXM was 42.8% and 57.1%, respectively. The infected nontreated mice recorded similar results as the mice treated with 2.66 mg/kg/day (Figure 4).

### 3.3. Profiles of IgM and IgG

**Figure 5:**
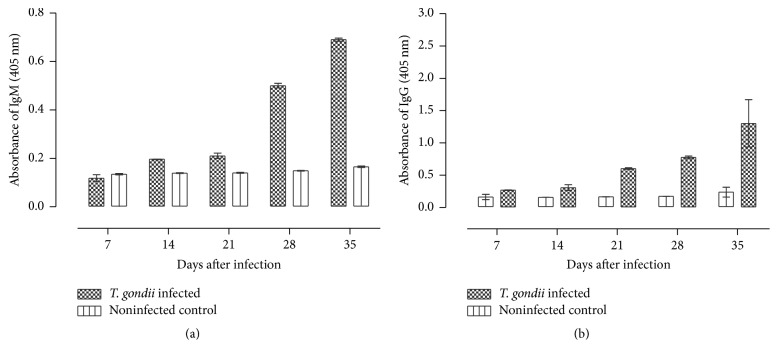
Profile of the IgM and IgG antibody levels in sera from mice intraperitoneally infected with* T. gondii*. Data represent the mean and standard deviations obtained from four mice per each time point.

The profiles of IgM and IgG antibody responses are shown in Figures 5(a) and 5(b), respectively. After* T. gondii* infection, the OD of IgM significantly (*P* < 0.05) increased from 0.12 (95%; CI: 0.089–0.15) at 7 dpi and peaked at 0.69 (95%; CI: 0.68–0.71) at 35 dpi. There was no corresponding increase in IgM titers in the noninfected nontreated mice. On the other hand, there was a significant (*P *< 0.05) increase in the OD of IgG rising from 0.27 (95%; CI: 0.26–0.28) at 7 dpi to 1.302 (95%; CI: 0.59–2.02) at 35 dpi. There was no corresponding increase in IgG levels in the noninfected nontreated mice.

**Figure 6:**
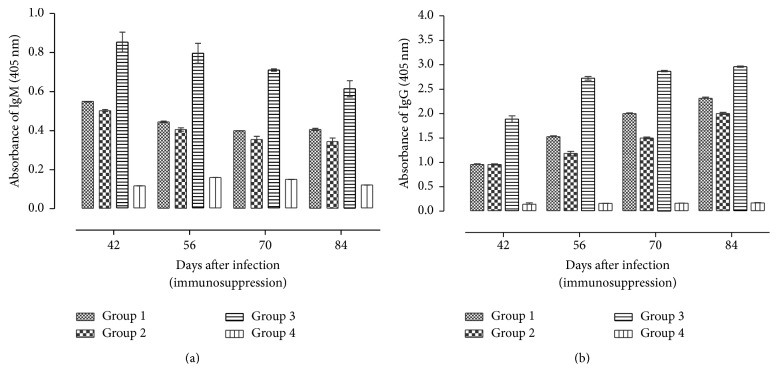
Profiles of the IgM (a) and IgG (b) antibodies in sera from mice intraperitoneally infected with* T. gondii* and later (42 dpi) treated with dexamethasone. Data represent the mean and standard deviations obtained from four mice per each time point: Group 1 = mice infected with* T. gondii* and treated with dexamethasone at 2.66 mg/kg/day; Group 2 = mice infected with* T. gondii* and treated with dexamethasone at 5.32/kg/day; Group 3 = mice infected with* T. gondii* and not treated with dexamethasone; Group 4 = noninfected control.

In the dexamethasone-treated mice, there was a significant (*P* < 0.001) decrease in IgM levels at time points between 42 and 84 days after infection (Figure 6(a)). The decline in the mice treated with 2.66 mg/kg/day was from 0.55 OD (95%; CI: 0.503–0.599) (42 dpi) to 0.41 OD (95%; CI: 0.29–0.42) (84 dpi) while the decline in the mice treated with 5.32 mg/kg/day was from 0.503 OD (95%; CI: 0.49–0.52) (42 dpi) to 0.34 OD (95%; CI: 0.29–0.38) (84 dpi). This shows that IgM levels decrease was associated with increased dose, although the differences were not significant (*P* > 0.05). The noninfected nontreated mice maintained significantly decreased levels of IgM compared to the infected nontreated and the infected treated mice (*P* < 0.001).

Following dexamethasone treatment, the levels of IgG maintained an upward trend in mice in all the groups (Figure 6(b)). The OD level of IgG in the mice treated with 2.66 mg/kg/day was 0.96 (95%; CI: 0.93–0.99) at 42 dpi and increased to 2.31 OD (95%; CI: 2.26–2.36) at 84 dpi. The OD level of IgG in the mice treated with 5.32 mg/kg/day was 0.95 (95%; CI: 0.93–0.97) at 42 dpi and increased to 2.004 O.D (95%; CI: 1.47–1.53) at 84 dpi. Significantly (*P* < 0.001) decreased levels of IgG were recorded in the treated mice compared to the infected nontreated at all time points after treatment. There was significant (*P* < 0.05) decrease in IgG antibody levels between treated mice with increase in dosage level. Overall, IgG levels were significantly (*P* < 0.05) higher compared to IgM at all postinfection time points.

### 3.4. Histological Changes in the Brains of* T. gondii*-Infected Mice Treated with Dexamethasone

**Figure 7:**
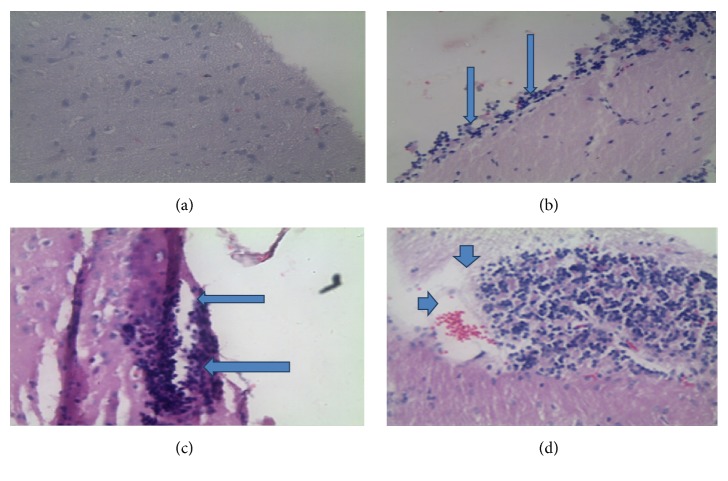
Histopathological findings in the brain of mice infected* T. gondii*. (a) Brain of uninfected mice. There are no alterations throughout the tissue. (b and c) Brain of infected mice. Increased number of lymphoid cells can be seen in the meninges (b) (arrows), suggesting the development of meningitis. (c) Perivascular cuffing by lymphoid infiltrations (arrows) along the meninges. (d) Lymphoid inflammation with zones of necrosis (arrow heads).

As shown in Figure 7, the lesions in the various groups of mice were characterized by diffuse infiltrates of mononuclear cells, glial nodules, vascular cuffing by lymphocytes, and focal mononucleated cell infiltrates in the meninges.

During the early stages of the infection (0–42 dpi) before DXM treatment, inflammatory changes in the brain were observed with the development of infection. From 7 dpi, the brain tissues from the infected mice presented with inflammatory lesions characterized mainly by lymphocyte infiltration into the brain meninges. This infiltration increased and, by 42 dpi, perivascular cuffing by lymphocytes was pronounced.

**Figure 8:**
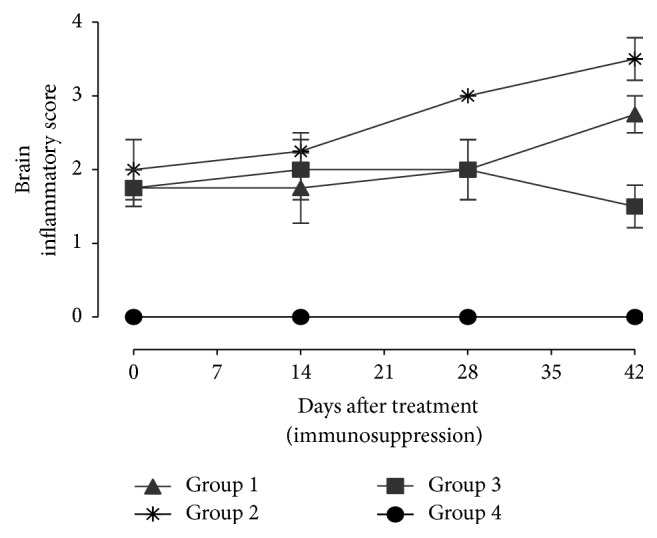
Inflammatory score in the brain of BALB/c mice infected with* T. gondii* after treatment with dexamethasone: Group 1 = mice infected with* T. gondii* and treated with dexamethasone at 2.66 mg/kg/day; Group 2 = mice infected with* T. gondii* and treated with dexamethasone at 5.32 mg/kg/day; Group 3 = mice infected with* T. gondii* and not treated with dexamethasone; Group 4 = noninfected control.

After dexamethasone treatment, there was an increased inflammatory response with increase in dosage level (Figure 8). Mice in the group treated with 2.66 mg/kg/day and 5.32 mg/kg/day of DXM had a minimal to moderate inflammatory changes including perivascular cuffs, meningitis, glial cell activation and focal necrosis, and rarefaction of the neuropil with occasional macrophage infiltration at 4 and 6 weeks after treatment.

Proportionately, brain tissue necrosis was observed in 14.3% and 28.5% of mice treated with 2.66 mg/kg/day and 5.32 mg/kg/day, respectively. No necrotic alterations were observed in the other groups. Gliosis was observed in 71.4% and 57.2% of brain tissues from mice treated with 2.66 mg/kg/day and 5.32 mg/kg/day, respectively, and all (100%) mice in the infected nontreated mice.

The proportion of mice with lymphocyte infiltration in the group treated with 2.66 mg/kg/day and 5.32 mg/kg/day and the infected nontreated group was 28.5%, 71.4%, and 100%, respectively. Numerous perivascular and leptomeningeal infiltrations of lymphoid cells and macrophages were observed, most often on the surface of the cerebral cortex and in the thalamus. Other types of pathology observed in the mice were formation of microglial nodules with surrounding astrocytosis. Increased intake of dexamethasone in mice showed a slight reduction in the severity of microgliosis and astrocytosis.

## 4. Discussion

The results of this study show that BALB/c mice can be used to develop a good model for toxoplasmosis. During the acute stage, the disease is characterized by tachyzoite proliferation, followed by a chronic stage, mainly characterized by the appearance of latent cysts within the central nervous system [21]. The clinical manifestations and the ensuing pathology are mainly influenced by strain of the parasite and immune status of the host [9]. The present study sought to determine the effect of administration of dexamethasone (DXM), an immunosuppressive drug, on pathogenesis of the disease in mice.

In this study, treated mice exhibited varied degrees of survival rates with higher mortalities being observed in mice given 2.66 mg/kg/day of DXM possibly due to elevated parasite burden in the brain. Higher tissue cysts numbers correlated with dose of DXM and the results are similar to that of a previous study [17]. The infected mice had signs of toxoplasmosis which has been reported earlier [7]. Clinical disease with typical locomotor signs of cerebral toxoplasmosis in DXM treated animals was associated with significantly shorter survival. It has been shown that immunosuppression of chronically infected mice results in the reactivation of a latent infection, leading to proliferation of tachyzoites [22].

The current study showed that mice infected with* T. gondii* developed pathology which was associated with diffuse infiltration of lymphocytes, glial nodules, vascular cuffing by lymphocytes, and gliosis. Other studies have shown that lymphocytes and plasma cells are the predominant cells in brains of patients having coinfection of HIV and toxoplasmosis [23]. In the current study, increased intake of dexamethasone resulted in increase in the extent of brain cellular inflammation and necrosis. This has also been observed in patients having clinical toxoplasmosis characterized by inflammation, to which microglia respond by forming nodules in attempts to contain the infection [24]. This was evident in this study and often this containment effort is simply ineffective due to the immunocompromised nature of the host. Human toxoplasmosis is normally associated with coagulative necrosis of infected cells and around the necrosis there are inflammatory cells, newly formed capillaries, edema, reactive astrocytes, and microglia [25]. The clinical effects of this uncontrolled infection are often dire.

The study showed that there was a decrease in the number of lymphoid cells in the brain, following treatment with DXM, suggesting the diminished immune response. Most infections by* T. gondii* are asymptomatic, but the risk of brain infection increases drastically in immunocompromised individuals. Immunosuppression of the host as in the case of immunosuppression caused by increasing DXM treatment results in uncontrolled release of bradyzoites during rupture of cysts in the brain of latently infected individuals. Subsequently, released bradyzoites convert into rapidly proliferating tachyzoites that may cause severe brain damage if left untreated [26].

DXM like the other corticosteroid drugs have long been known to cause immunosuppression through lymphocytopenia, monocytopenia, and shrinkage of the spleen [27]. In these immunosuppressed hosts, the infection in the central nervous system can lead to severe pathogenesis which is associated with toxoplasmosis as was the case in this study. A comparison done with other studies involving immunosuppressed humans has shown that cerebral toxoplasmosis is among the most frequent CNS pathologies and as many as one-third of all* T. gondii*-infected HIV-positive patients not treated with antiretroviral therapy may develop toxoplasmic encephalitis [28].

Sera IgG and IgM in the present study increased immediately after* T. gondii* infection and these findings are similar to those reported by Kang et al. [8]. Indeed, antibodies levels are often used in diagnosis of toxoplasmosis [29]. Following dexamethasone treatment, serum IgM titers of the* Toxoplasma* infected and treated mice were depressed significantly, whereas serum IgG titers were not significantly altered. Previous studies have shown that sera from mice acutely infected with toxoplasmosis had higher IgM levels than those from chronically infected mice [30]. In humans, it has been shown that* Toxoplasma* IgG antibody levels are associated with onset of schizophrenia and epilepsy [31, 32]. It is postulated that proliferation of tachyzoites in the brain during the chronic stage of infection may be involved in the etiology of schizophrenia and cryptogenic epilepsy [33].

## 5. Conclusions

The current BALB/c model which used a local isolate of* T. gondii* showed pathological and immunological profiles with some similarities to that reported in patients having chronic toxoplasmosis. The model should facilitate investigation of the pathobiology, diagnosis, and treatment of toxoplasmosis, although the anti-inflammatory effects of the drug must be put into consideration.
